# Catalysis of linear alkene metathesis by Grubbs-type ruthenium alkylidene complexes containing hemilabile α,α-diphenyl-(monosubstituted-pyridin-2-yl)methanolato ligands

**DOI:** 10.3762/bjoc.15.19

**Published:** 2019-01-22

**Authors:** Tegene T Tole, Johan H L Jordaan, Hermanus C M Vosloo

**Affiliations:** 1 Research Focus Area for Chemical Resource Beneficiation, Catalysis and Synthesis Research Group, North-West University, Hoffmann Street, 2531 Potchefstroom, South Africa; 2 Department of Chemistry, College of Natural and Computational Sciences, Hawassa University, Hawassa, Ethiopia

**Keywords:** Grubbs-type precatalyst, hemilabile, 1-octene metathesis, pyridinyl-alcoholato ligand

## Abstract

Four new Grubbs-type precatalysts [RuCl(H_2_IMes)(O^N)(=CHPh)], where [O^N = α,α-diphenyl-(3-methylpyridin-2-yl)methanolato, α,α-diphenyl-(4-methylpyridin-2-yl)methanolato, α,α-diphenyl-(5-methylpyridin-2-yl)methanolato and α,α-diphenyl-(3-methoxypyridin-2-yl)methanolato] were synthesized and tested for their activity, stability and selectivity in the 1-octene metathesis reaction. Overall the precatalysts showed good activity and high stability for the metathesis of 1-octene at temperatures above 80 °C and up to 110 °C. Selectivities towards the primary metathesis products, i.e., 7-tetradecene and ethene, above 85% were obtained with all the precatalysts at 80 and 90 °C. High selectivities were also observed at 100 °C for the 4-Me- and 3-OMe-substituted precatalysts. With an increase in temperature an increase in isomerisation products and secondary metathesis products were observed with the latter reaching values >20% for the 3-OMe- and 3-Me-substituted precatalysts at 110 and 100 °C, respectively. All the precatalysts exhibits first-order kinetics at 80 °C with the 3-substituted precatalysts the slowest. The behaviour of the 3-substituted precatalysts can be attributed to electronic and steric effects associated with the adjacent bulky phenyl groups.

## Introduction

**Figure 1:**
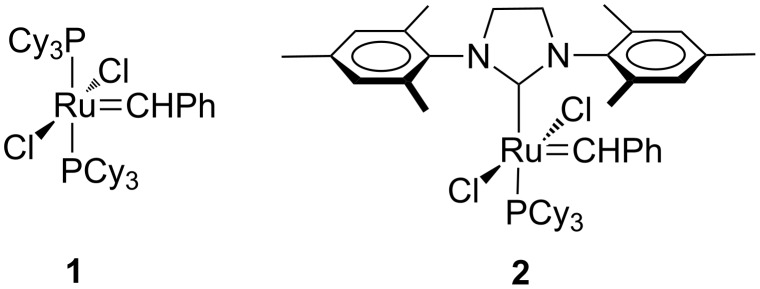
Structures of Grubbs 1 (**1**) and 2 (**2**) precatalysts.

The alkene metathesis reaction is now well established as a powerful synthetic tool in organic and polymer chemistry [1–2]. The development of metal alkylidene precatalysts based on ruthenium, starting with the so-called Grubbs 1 (**1**) and 2 (**2**) metal carbenes, played a major role to extend the versatility of the reaction including the application of these in industrial processes (Figure 1). Of course, the role of the so-called Schrock metal carbenes based on tungsten and molybdenum should not be ignored in the success story of the alkene metathesis reaction but it is not the focus of this article.

**Scheme 1:**
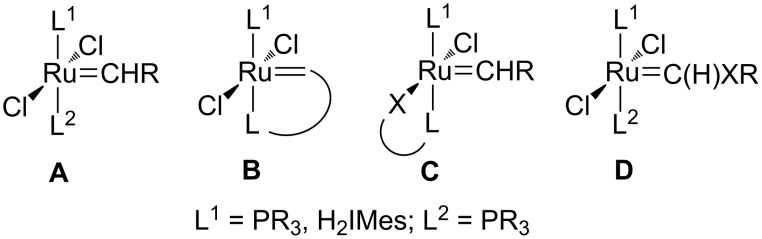
Design concepts for ruthenium alkylidene precatalysts [3].

The large number of ruthenium alkylidene precatalysts that has been developed is based on the design concepts illustrated in Scheme 1 [3]. The design concept **C** is of interest because of the potential hemilabile nature and latent metathesis activity of these complexes [4]. Of particular interest to us are the ruthenium alkylidene complexes containing the pyridinyl alcoholato bidentate ligands investigated by a number of research groups [5].

**Figure 2:**
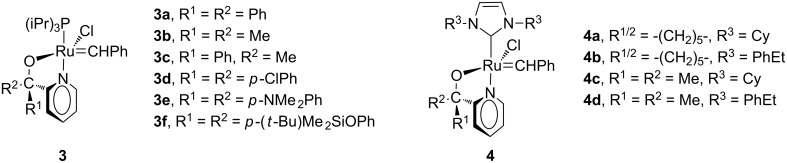
Structures of Grubbs 1-type (**3**) and 2-type (**4**) pyridinyl-alcoholato precatalysts.

The pyridinyl alcohol found its way to the Grubbs-type complexes from research by Van Der Schaaf and co-workers on the Schrock-type analogues [6–7]. Grubbs 1-type complexes **3a**–**f** (Figure 2) were used to catalyse the ring-closing metathesis (RCM) of dialkenes, ring-opening metathesis polymerisation (ROMP), isomerisation of alkenes and cross-metathesis (CM) of alkenes [7]. The complexes were synthesized by reacting the lithium salts of the corresponding pyridinyl alcohols with [RuCl_2_(=CHC_6_H_5_)(P(iPr_3_))_2_]. These complexes catalysed inter alia the cyclisation of hex-5-enyl undec-10-enoate to oxacyclohexadec-11-en-2-one (50% at 60 °C in toluene) and the ROMP of dicyclopentadiene. They were also able to immobilise these Grubbs 1-type precatalysts using dendritic pyridinyl alcohols [8]. These complexes catalysed the RCM reaction (at 80 °C) of diethyl diallylmalonate with 100% conversion after 30 min, results comparable to the unimolecular catalyst.

Denk et al. [9] synthesised *N*-heterocyclic (NHC) ruthenium alkylidene complexes containing pyridinyl-alcoholato ligands (**4**). These complexes were tested as precatalysts at different temperatures in the ROMP of norbornene and cyclooctene. Oligomers were obtained at room temperature in the presence of **4a** and **4d**, while **4b** and **4c** yielded polymers. At 60 °C, ROMP was observed with norbornene (98–100%) and cyclooctene (72–80%) in the presence of **4**.

**Figure 3:**
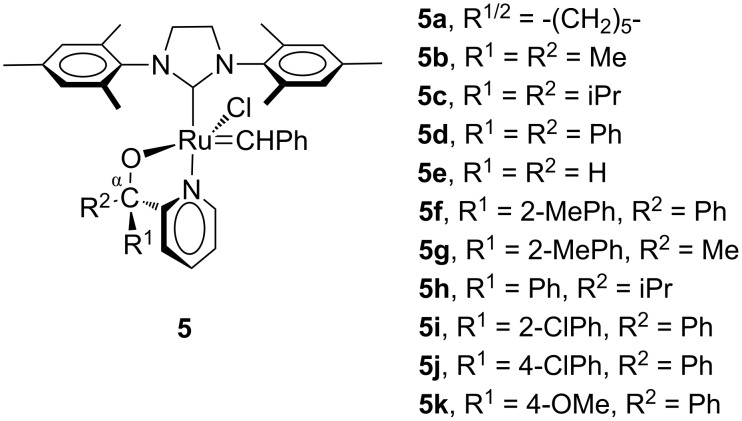
Structures of Grubbs 2-type (**5**) pyridinyl-alcoholato precatalysts.

We investigated a number of Grubbs 1- and Grubbs 2-type (**5**) metal carbenes with pyridinyl alcoholato ligands for the 1-octene metathesis reaction (Figure 3) [10–14]. The incorporation of pyridinyl-alcoholato ligands in the Grubbs-type precatalysts has shown an increase in the thermal stability, activity and lifetime of the precatalysts when compared to **1** and **2** [10]. The pyridinyl-alcoholato Grubbs 2-types exhibited higher activities and selectivities than the Grubbs 1-types and were investigated in more detail. It is clear from the results that the chelating ability of the pyridinyl alcoholato ligands combined with the NHC ligand is responsible for the activity and improved stability of the precatalyst at high temperatures. In general **5d** performed the best in the 1-octene metathesis reactions when compared to complexes **5a**–**c** and **5e**–**h**. The catalytic performance could be further tuned by the incorporation of an electron-donating (e.g., OMe, **5k**) or electron-withdrawing (e.g. Cl, **5i** and **5j**) group at the 2- or 4-position of one of the α-phenyl groups of **5d** [14]. At 80–110 °C these complexes showed improved catalytic performance in the metathesis of oct-1-ene. At 110 °C complex **5k**, with 96% conversion and 95% selectivity towards the primary metathesis products tetradec-7-ene and ethene, outperformed the other complexes. In a computational study the improved catalytic performance was attributed to strengthening of the Ru–N bond due to steric repulsion between the substituted phenyl group and the NHC ligand [14]. An 8-quinolinolate Grubbs 2-type derivative, patented by Slugovc and Wappel [15] for use in ROMP reactions, was found to be inactive (<1% conversion) for 1-octene metathesis at 60 °C [12].

Schachner et al. [16] evaluated the catalytic activity of **5b**, **5d** and related complexes for the ROMP of cyclooctene, CM of hex-5-enyl acetate with dec-5-ene and the RCM of hex-5-en-1-yl undec-10-enoate. Superior (CM, RCM) to moderate (ROMP) activities were observed for most of these precatalysts. An interesting result was the very high affinity (“stickiness”) to untreated, unmodified and commercially available chromatography-grade silica. This was exploited further by Cabrera et al. [17–18] when **5b** and related complexes were investigated as heterogeneous precatalysts in biphasic RO-RCM and CM reactions. The substrate and catalyst were adsorbed on a thin layer silica plate and developed in EtOAc/hexane (1:7 v/v) for the CM of methyl 9-dodecene and in hexane for the RO-RCM of *cis*-cyclooctene.

**Figure 4:**
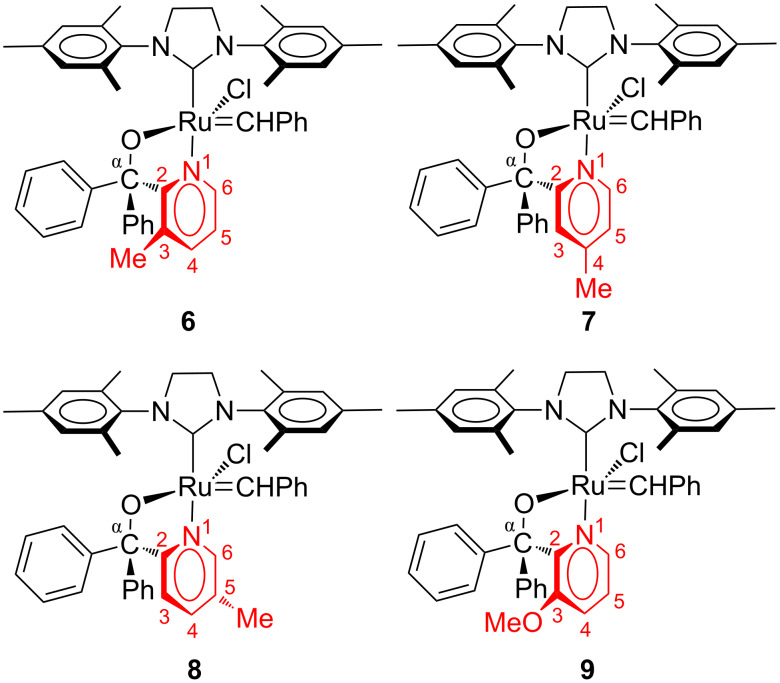
Structures of pyridinyl-substituted Grubbs 2-type pyridinyl-alcoholato precatalysts.

The above-mentioned studies clearly illustrate the versatility and use of ruthenium alkylidene complexes with pyridinyl-alcoholato ligands. In principle these studies had one approach in common concerning the pyridinyl-alcoholato ligand, and that was to focus on substituents on the α-carbon of the ligand. To our knowledge, there are no reports on investigations of electronic and/or steric effect(s) of pyridinyl substituents on the chelation efficiency of pyridinyl alcoholato ligands, and subsequently its metathesis activity. Therefore, in this paper, we investigated the influence of a monosubstituent on the pyridinyl moiety on the 1-octene metathesis activity of a Grubbs 2-type precatalyst with an α,α-diphenyl methanolato ligand. For the synthesis of the pyridinyl methanol compounds, commercially available substituted bromopyridines were reacted with benzophenone followed by a reaction of the lithiated alcohol with **2**. Four new ruthenium alkylidene complexes, i.e., **6**–**9** (Figure 4), were successfully obtained and investigated as precatalyst in 1-octene metathesis in the temperature range 40–110 °C. The stability, selectivity and turnover frequency (TOF) of **2** increased upon substituting Me and OMe groups on the various positions of the pyridine ring of the pyridinyl-alcoholato ligands at high temperatures (80–110 °C). The increase in stability is attributed to the electronic and steric influence of the Me and OMe groups on Ru–N chelation. The activity of the precatalysts also showed a significant improvement upon increasing the reaction temperature from 40 to 110 °C. The increase in the activity of the precatalysts is relatively low in the 40–60 °C range, but a high activity difference is observed upon increasing the temperature in 10 °C intervals between 70 and 110 °C.

## Results and Discussion

A mixture of products, summarised in Table 1, is obtained during the metathesis of 1-octene, i.e., primary metathesis products (PMPs), isomerisation products (IPs) and secondary metathesis products (SMPs). The PMPs, 7-tetradecene (*cis* and *trans*) and ethene, forms as a result of the self-metathesis (SM) of 1-octene. Simultaneously 1-octene is isomerised to 2-, 3- and 4-octene (IPs). The subsequent SM and CM reactions of the internal alkenes yield alkenes (*cis* and *trans*) in the C_3_–C_13_ range (SMPs).

**Table 1:** Mixture of products formed during 1-octene metathesis in the presence of ruthenium alkylidene precatalysts.

Reaction	Substrate^a^	Products^a^	Abbrev.

primary metathesis			PMPs
self-metathesis	C=C_7_	C=C + C_7_=C_7_	
isomerisation	C=C_7_	C_2_=C_6_ + C_3_=C_5_ + C_4_=C_4_	IPs
secondary metathesis			SMPs
cross metathesis	C=C_7_ + C_2_=C_6_	C_2_=C_7_ + C=C_6_ + C=C_2_ + C_6_=C_7_	
self-metathesis	C_2_=C_6_	C_2_=C_2_ + C_6_=C_6_	

^a^Geometrical isomers and hydrogens are not shown for simplicity.

All the reactions were followed by GC at regular sampling intervals until 540 min. Because the observed formation of IPs is mostly below 2% and never above 4% it is also not shown in the figures.

### Effect of the reaction temperature

**Figure 5:**
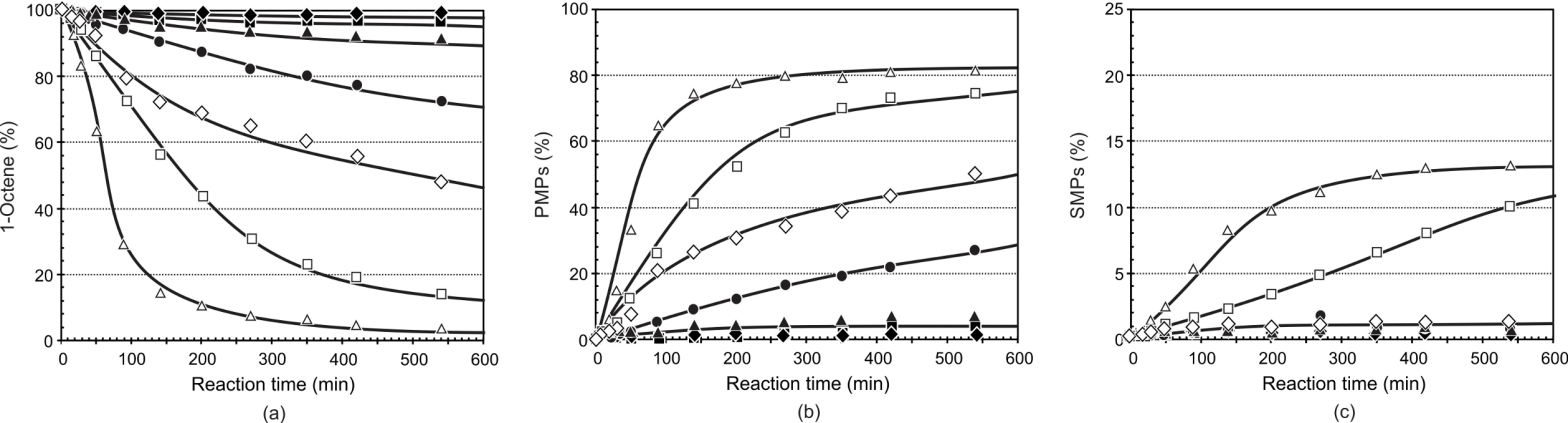
The influence of the reaction temperature on the (a) conversion of 1-octene, (b) formation of PMPs and (c) formation of SMPs using precatalyst **7** (Ru/1-octene = 1:9000). [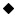 40 °C, ■ 50 °C, ▲ 60 °C, ● 70 °C, ◊ 80 °C, □ 90 °C, ∆ 100 °C].

The results of the metathesis of 1-octene at temperatures 40–100 °C are presented in Figure 5 and Table 2 for precatalyst **7**. The rate of self-metathesis of 1-octene showed an increase upon raising the reaction temperature from 40 to 100 °C. The metathesis reaction is insignificant at lower temperatures (40 to 60 °C). Upon raising the temperature beyond 70 °C, an increase in the reaction rate was observed, resulting in a significant increase in 1-octene conversion greater than 80% at 90 °C after 540 min. Increasing the temperature further to 100 °C showed a dramatic increase in the metathesis reaction rate and a high PMPs formation are observed at 100 °C (>85% after ca*.* 200 min).

**Table 2:** Summary of the catalytic performance of precatalyst **7** at different temperatures (Ru/1-octene molar ratio 1:9 000, 420 min).

Entry	Temp. [°C]	Conv.^a^	PMPs^a^	SMPs^a^	IPs^a^	S^b^	TON^c^	TOF^d^

1	40	0.8	0.2	0.2	0.4	23	18	0.07 × 10^−2^
2	50	2.1	0.9	0.4	0.8	44	81	0.32 × 10^−2^
3	60	7.2	5.8	1.1	0.3	80	522	2.07 × 10^−2^
4	70	22.3	21.2	0.9	0.2	95	1908	7.57 × 10^−2^
5	80	44.4	43.1	1.1	0.2	97	3879	15.39 × 10^−2^
6	90	81.4	72.4	8.0	1.0	89	6516	25.86 × 10^−2^
7	100	95.2	83.1	11.0	1.1	87	7479	29.68 × 10^−2^

^a^Conversion or yield in mol %; ^b^S (selectivity) in percent toward PMPs; ^c^TON (turnover number) = [%PMPs × (Oct/Ru)]/100; ^d^TOF (turnover frequency) = TON/time in s.

The formation of PMPs did not equilibrate within 540 min for the temperature range 50 to 80 °C; however, its formation equilibrated at ca*.* 400 min at 90 °C and ca. 200 min at 100 °C. This shows that **7** is stable at high temperatures, with moderate to very good PMPs (ca. 25–80%) formation. On the other hand, the formation of SMPs (ca. 0.2–2.0%) and IPs (ca. 0.2–0.5%) is negligible in the range 40–80 °C, while a more significant amount is formed at temperatures greater than 90 °C (ca. 10–14% SMPs and 1.7–2.1% IPs) after 540 min.

Table 2 summarises the overall catalytic performance of **7** at 420 min. In this period it can be seen that the PMPs and SMPs formation, TON, and TOF of the precatalyst show a direct relationship with temperature. The highest PMPs formation is observed for the temperature changes from 80 to 90 °C (29.3%) and the least for 40 to 50 °C (0.7%) at 420 min.

The selectivity towards PMPs showed a dramatic increase upon increasing the temperature from 40 to 80 °C (23–97%); however, it showed a decrease going from 80 (97%) to 90°C (89%), and then to 100 °C (87%). An overall assessment of the results show that at 80 °C the catalyst showed a high selectivity for PMPs with a negligible amount of SMPs and IPs. Although the activity of the precatalyst increased a great deal at 90 and 100 °C, the selectivity for PMPs decreased as a result of the high amount of SMPs and IPs formation. The TOF increased significantly as a result of increasing the temperature. The highest TOF increase was observed upon increasing the temperature from 80 to 90 °C. Generally, precatalyst **7** showed very good activity, selectivity and stability at high temperatures.

**Figure 6:**
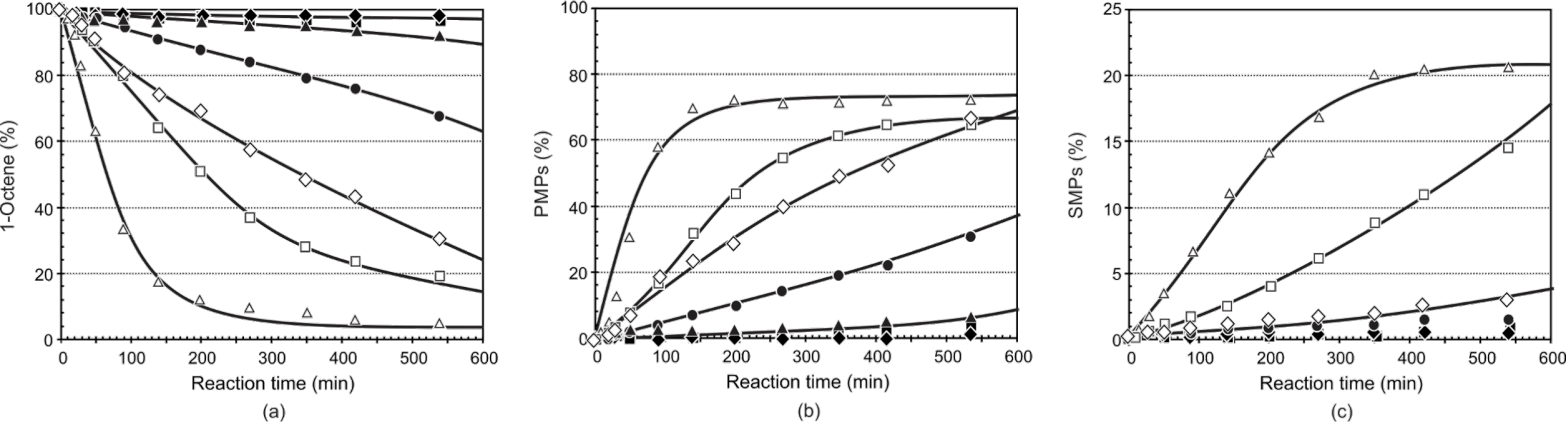
The influence of the reaction temperature on the (a) conversion of 1-octene, (b) formation of PMPs and (c) formation of SMPs using precatalyst **8** (Ru/1-octene = 1:9000). 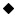 40 °C, ■ 50 °C, ▲ 60 °C, ● 70 °C, ◊ 80 °C, □ 90 °C, ∆ 100 °C].

The results of the metathesis of 1-octene at temperatures 40–100 °C are presented in Figure 6 and Table 3 for precatalyst **8**. A similar overall trend for **8** is observed, i.e., very low reaction rates at temperatures below 60 °C with a rapid increase in reaction rates above 70 °C resulting in 1-octene conversions above ca. 70% after 540 min. Although the formation of PMPs equilibrated quickly at ca. 70% after ca. 150 min at 100 °C and at ca. 65% only after ca. 400 min for 90 °C it did not equilibrate at 80 °C even after 540 min.

**Table 3:** Summary of catalytic performance of precatalyst **8** at different temperatures (Ru/1-octene molar ratio 1:9 000, 420 min).

Entry	Temp. [°C]	Conv.^a^	PMPs^a^	SMPs^a^	IPs^a^	S^b^	TON^c^	TOF^d^

1	40	1.5	0.8	0.3	0.4	56	72	0.28 × 10^−2^
2	50	2.9	1.4	0.6	0.9	49	126	0.50 × 10^−2^
3	60	5.3	4.5	0.7	0.1	85	405	1.61 × 10^−2^
4	70	24.0	22.7	1.1	0.2	94	2043	8.11 × 10^−2^
5	80	65.4	60.5	4.1	0.8	92	5445	21.61 × 10^−2^
6	90	77.3	65.0	11.0	1.3	84	5850	23.21 × 10^−2^
7	100	93.8	73.0	19.0	1.8	78	6570	26.07 × 10^−2^

^a^Conversion or yield in mol %; ^b^S (selectivity) in percent toward PMPs; ^c^TON (turnover number) = [%PMPs × (Oct/Ru)]/100; ^d^TOF (turnover frequency) = TON/time in s.

The formation of SMPs is very low (below 4%) for **8** in the temperature range 40–80 °C after 540 min, while it is relatively high at 14 and 21% at 90 and 100 °C, respectively. In the same period the formation of IPs remained below 3% even at the high temperatures. At 100 °C and 540 min, a larger amount of SMPs is formed for precatalyst **8** than that of **7**.

Table 3 summarises the overall catalytic performance of precatalyst **8** at 420 min. The PMPs and SMPs formation, TON and TOF all show a direct relationship with temperature. Precatalysts **7** and **8** share similarities in having the same temperature range for the highest PMPs formation, i.e., 70 to 80 °C at 420 min. The biggest difference, however, is observed for **8** (37%). Relatively higher SMPs are formed for **8** (11%, 19%) than that of **7** (8%, 11%) at 90 and 100 °C, respectively. The relatively low PMPs formation of **8** compared to that of **7** is due to the relatively high SMPs and IPs formations with precatalyst **8**. The IPs formation in **8** follows a similar pattern to that of **7**, i.e., it showed an increase upon increasing the temperature from 40 to 50 °C, followed by a decrease from 50 to 60 °C and then an increase from 60 to 100 °C. The selectivity in **8** increased upon increasing the temperature from 40 to 70 °C, and then showed a decrease from 70 to 100 °C.

A maximum selectivity for **8** is observed at 70 °C (94%) (see Table 3, entry 4) and for that of **7** at 80 °C (97%) (see Table 2, entry 5). Generally, precatalyst **7** showed a better selectivity compared to that of **8** at 420 min. Although the TOF of **8** is in direct relation with temperature, it follows the following order upon comparing with **7**; **8** > **7** at 40 and 50 °C (see Table 2 and Table 3, entries 1–7), at 60 °C **7** > **8** (see Table 2 and Table 3, entry 3), at 70 and 80 °C **8** > **7** (see Table 2 and Table 3, entries 4 and 5) and **7** > **8** at 90 and 100 °C (see Table 2 and Table 3, entries 6 and 7).

Summarising the comparisons of precatalysts **7** and **8,** it is noted that precatalyst **7** showed better activity, selectivity and stability in the 60–100 °C temperature range, except for 80 °C, at 420 min. It also showed higher TOF at 60, 90 and 100 °C at 420 min. According to a DFT study by Getty et al. [19] the more positively charged the Ru, the slower the initiation rate of the catalyst. The calculated Mulliken atomic charge of Ru in **7** (0.934) is less positive than in **8** (0.976).

**Figure 7:**
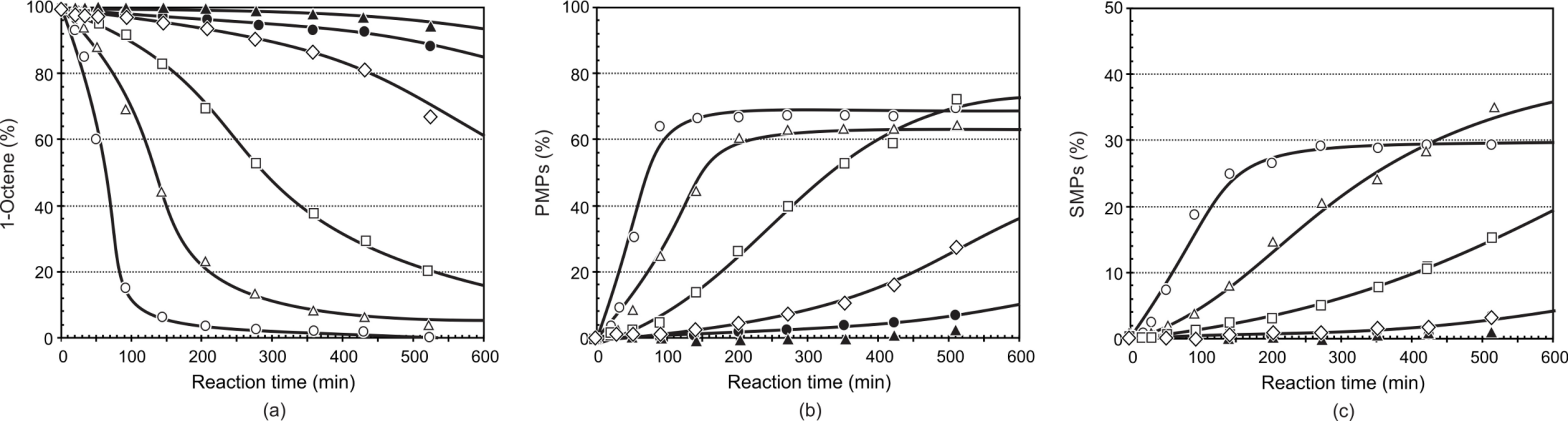
The influence of the reaction temperature on the (a) conversion of 1-octene, (b) formation of PMPs and (c) formation of SMPs using precatalyst **6** (Ru/1-octene = 1:9000). [▲ 60 °C, ● 70 °C, ◊ 80 °C, □ 90 °C, ∆ 100 °C, ○ 110 °C].

**Figure 8:**
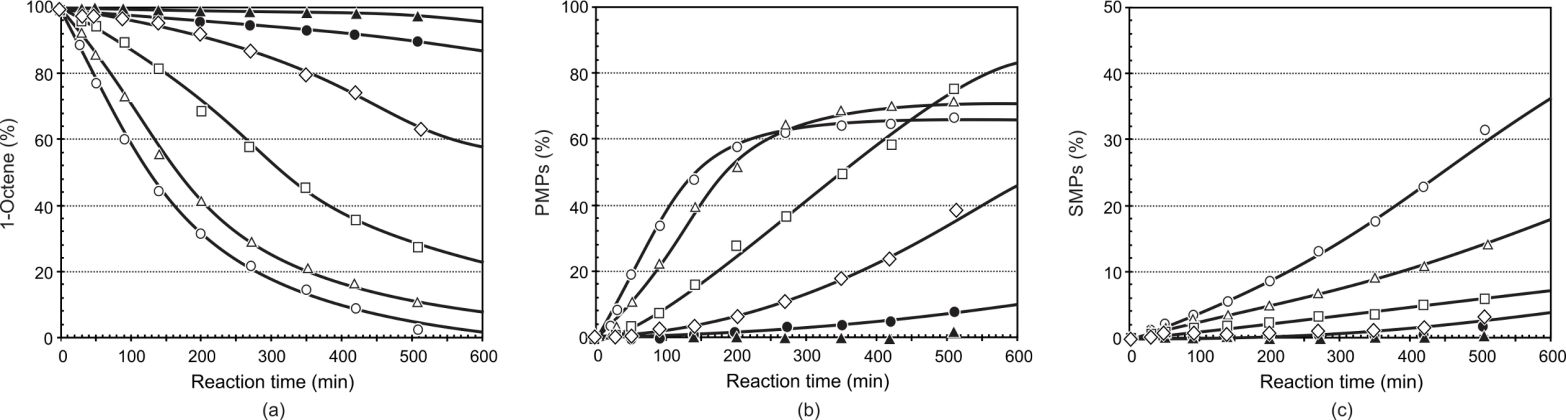
The influence of the reaction temperature on the (a) conversion of 1-octene, (b) formation of PMPs and (c) formation of SMPs using precatalyst **9** (Ru/1-octene = 1:9000). [▲ 60 °C, ● 70 °C, ◊ 80 °C, □ 90 °C, ∆ 100 °C, ○ 110 °C].

The results of the metathesis of 1-octene at temperatures of 60 to 110 °C are presented in Figure 7 and Figure 8 for precatalysts **6** and **9**, respectively. Because of their low activity and high stability, the metathesis reactions were done between 60 and 110 °C. Metathesis of 1-octene by the 3-Me-substituted precatalyst **6** showed an increase in the activity of the precatalyst upon increasing the temperature from 60 to 110 °C. A large increase in the rate of the metathesis reaction is observed upon increasing the temperature from 80 to 90 °C. Although the activity of the precatalyst has shown an increase upon increasing the temperature from 60 to 110 °C, a very high (ca. 45%) increase in the PMPs formation is observed upon increasing the temperature from 80 to 90 °C at 540 min. The PMPs formation did not equilibrate at 90 °C and this shows the stability of precatalyst **6** at high temperatures. The PMPs formation, however, equilibrated from ca. 270 min at 100 °C and ca. 140 min at 110 °C. At 80 °C, the activity of precatalyst **6** (ca. 25%) is very low compared to precatalysts **7** (ca. 50%) and **8** (ca. 70%) during the course of PMPs formation, at 540 min. Generally, in the first 100 to 300 minutes, the rate of formation of PMPs increases dramatically and slows down afterwards in the temperature range of 90 to 110 °C. A similar trend is observed during the course of SMPs formation. Significant amounts (16–36%) of SMPs are formed by **6** for temperatures 90 to 110 °C, while negligible amounts (1.3–3.6%) of SMPs are formed from 60 to 80 °C. Large amounts of SMPs (ca. 36%) and IPs (5%) were formed by **6** at 540 min at 100 °C. A comparison of precatalysts **6**, **7** and **8** with regard to SMPs and IPs formation at 100 °C and 540 min shows a decreasing order of **6** > **8** > **7**. Generally, a relatively large amount of IPs is formed by precatalyst **6**. Although the rate of PMPs formation is slow at 80 °C, high selectivity and stability are attained at this temperature for precatalyst **6,** similar to precatalysts **7** and **8**. The highest difference in the PMPs formation (ca. 45%) is observed between 80 and 90 °C. Although the SMPs formation has shown a direct relationship with temperature, the formation of high SMPs (16–36%) from 90 to 110 °C limited the PMPs formation to only a maximum of ca. 68% at 540 min. The contribution of the IPs formation in limiting the formation of PMPs is not negligible as a result of the relatively high (ca. 3.5%) IPs formation. The SMPs formation increased approximately six-fold upon increasing the temperature from 80 to 90 °C, which was then followed by approximately a two-fold increase upon increasing the temperature from 90 to 100 °C.

Table 4 presents the overall catalytic performance of precatalyst **6** at 420 min. At 420 min, 1-octene conversion is 98.0% (110 °C), 93.0% (100 °C), 70.5% (90 °C), 19.4% (80 °C), 6.8% (70 °C) and 2.5% (60 °C). This shows the dramatic increase of the catalytic activity upon increasing the temperature. An investigation of the PMPs formation reveals a huge 66% increase for the PMPs formation upon increasing the reaction temperature from 60 to 110 °C. The IPs formation, on the other hand, increased two-fold upon increasing the temperature from 60 to 70 °C, 80 to 90 °C and 90 to 100 °C.

**Table 4:** Summary of catalytic performance of precatalyst **6** at different temperatures (Ru/1-octene molar ratio 1:9000, 420 min).

Entry	Temp. [°C]	Conv.^a^	PMPs^a^	SMPs^a^	IPs^a^	S^b^	TON^c^	TOF^d^

1	60	2.5	1.0	0.9	0.6	41	93	0.36 × 10^−2^
2	70	6.8	4.5	1.0	1.3	66	406	1.61 × 10^−2^
3	80	19.4	16.4	2.3	0.7	85	1479	5.86 × 10^−2^
4	90	70.5	59.0	10.3	1.2	83	5310	21.07 × 10^−2^
5	100	93.0	61.3	28.5	3.9	66	5514	21.88 × 10^−2^
6	110	98.0	67.2	28.8	2.1	69	6051	24.01 × 10^−2^

^a^Conversion or yield in mol %; ^b^S (selectivity) in percent toward PMPs; ^c^TON (turnover number) = [%PMPs × (Oct/Ru)]/100; ^d^TOF (turnover frequency) = TON/time in s.

The selectivity toward PMPs (67.2%) and the SMPs (28.8%) are relatively high at 110 °C. The TOF is also directly related to the reaction temperature. The TOF of precatalyst **6** are generally lower than for precatalysts **7** and **8**. This, therefore, shows the relatively high stability of precatalyst **6** compared to those of **7** and **8**. As a result of having a more positive Ru charge, precatalyst **6** showed a low initiation rate. This is also in agreement with the DFT study of Getty et al. [19], i.e., precatalyst **6** (0.988) has more positive Mulliken’s atomic charge on Ru than both **7** (0.934) and **8** (0.976). Its high activity at 110 °C with 69% selectivity is, however, remarkable for linear alkene metathesis catalysed by ruthenium alkylidene precatalysts.

The 3-OMe-substituted precatalyst **9** showed a negligible activity for the metathesis of 1-octene at 60 °C (Figure 8), similar to the 3-Me-substituted precatalyst **6**. The overall activity of the precatalyst, however, showed a significant increase upon increasing the temperature from 60 to 110 °C. In a similar way to that of **6**, the largest increase in the activity of the precatalyst is observed upon increasing the temperature from 80 to 90 °C (ca. 38%) at 420 min. The activity of the catalyst showed a small difference between 100 and 110 °C, on the overall metathesis reaction.

During the course of PMPs formation, high catalytic activity for **9** is observed within 200 min at temperatures above 90 °C (ca. 60%), while the activity of the precatalyst showed a dramatic increase from 70 to 90 °C after ca. 500 min, similar to that of **6** (see Figure 7b). For both **6** and **9** the highest PMPs (>60%) is observed from 90 to 110 °C after 420 min. The rate of formation of SMPs is very high for **9** at 110 °C within 420 min. This is the reason for the decrease in the formation of PMPs from 71% at 100 °C to 64.2% at 110 °C.

Table 5 presents the overall catalytic performance of precatalyst **9** at 420 min. Firstly, PMPs formation increased from 0.5 to 71% when increasing the temperature from 60 to 100 °C; however, from 100 to 110 °C the PMPs yield decreased from 71 to 64.2%. The reason for this is the formation of a very large amount of SMPs (23.2%) and IPs (2.6%), which is, more than twice the amount at 100 °C. Similarly, the selectivity and TOF showed a decrease when going from 100 to 110 °C.

**Table 5:** Summary of catalytic performance of precatalyst **9** at different temperatures (Ru/1-octene molar ratio 1:9000, 420 min).

Entry	Temp. [°C]	Conv.^a^	PMPs^a^	SMPs^a^	IPs^a^	S^b^	TON^c^	TOF^d^

1	60	1.1	0.5	0.3	0.3	43	45	0.18 × 10^−2^
2	70	7.3	5.3	1.1	0.9	73	477	1.89 × 10^−2^
3	80	25.7	24.0	1.4	0.3	93	2160	8.57 × 10^−2^
4	90	64.0	58.3	5.1	0.6	91	5247	20.82 × 10^−2^
5	100	83.3	71.0	11.1	1.2	85	6390	25.36 × 10^−2^
6	110	90.0	64.2	23.2	2.6	71	5778	22.93 × 10^−2^

^a^Conversion or yield in mol %; ^b^S (selectivity) in percent toward PMPs; ^c^TON (turnover number) = [%PMPs × (Oct/Ru)]/100; ^d^TOF (turnover frequency) = TON/time in s.

The selectivity showed a dramatic (50%) increase upon increasing the temperature from 60 (43%) to 80 °C (93%) and then begins to decrease to 91% (at 90 °C), 85% (at 100 °C) and finally to 71% (at 110 °C). Although the catalyst showed significant stability and very good (71%) selectivity at 110 °C, it is advisable not to go beyond 100 °C, as the formation of SMPs and IPs doubled that will affect the overall PMPs yield. It is also worthwhile to note the decrease in the turnover frequency at 110 °C.

Similar to the Me-substituted precatalysts **6** (85%) (see Table 4, entry 3), **7** (97%) (see Table 2, entry 5) and **8** (92%) (see Table 3, entry 5), precatalyst **9** showed high selectivity (93%) towards PMPs and good stability at 80 °C after 420 min. It is also observed from the results that precatalyst **9** showed relatively high selectivity (91%) for PMPs and good activity, higher than its methyl counterparts **6** (83%) (see Table 4, entry 4), **7** (84%) (see Table 2, entry 6) and **8** (89%) (see Table 3, entry 6), at 90 °C after 420 min.

A general comparison of the overall performance of the precatalysts, in terms of PMPs, SMPs, IPs, selectivity, TON and TOF, exhibits the decreasing order of **7** > **9** > **8** > **6** at 60, 90 and 100 °C. The order, however, changes at 80 °C to **8** > **7** > **9** > **6** and at 70 °C to **7** 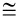 **8** > **9** > **6**. In all cases, the small amounts of SMPs and IPs are positive for the application of these systems at higher temperatures. Overall precatalyst **7** performed the best at all temperatures (except at 80 °C). In an attempt to understand the significance of these results, DFT calculations were performed on the precatalysts.

**Figure 9:**
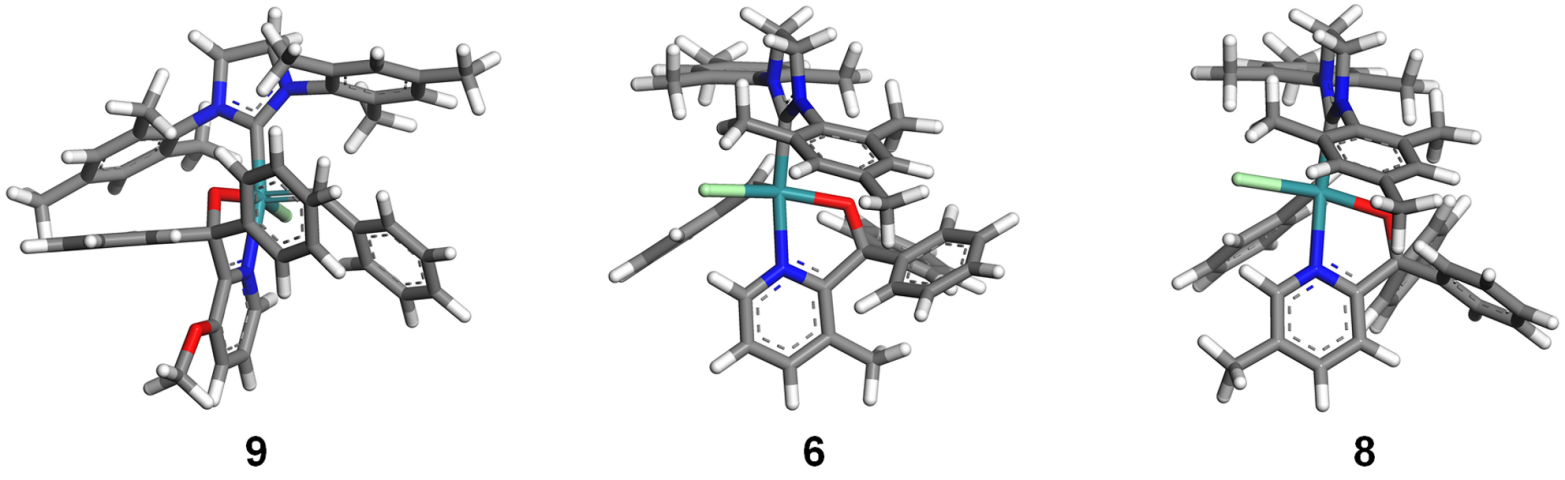
Geometry-optimised structures of precatalyst **9**, **6** and **8**.

**Figure 10:**
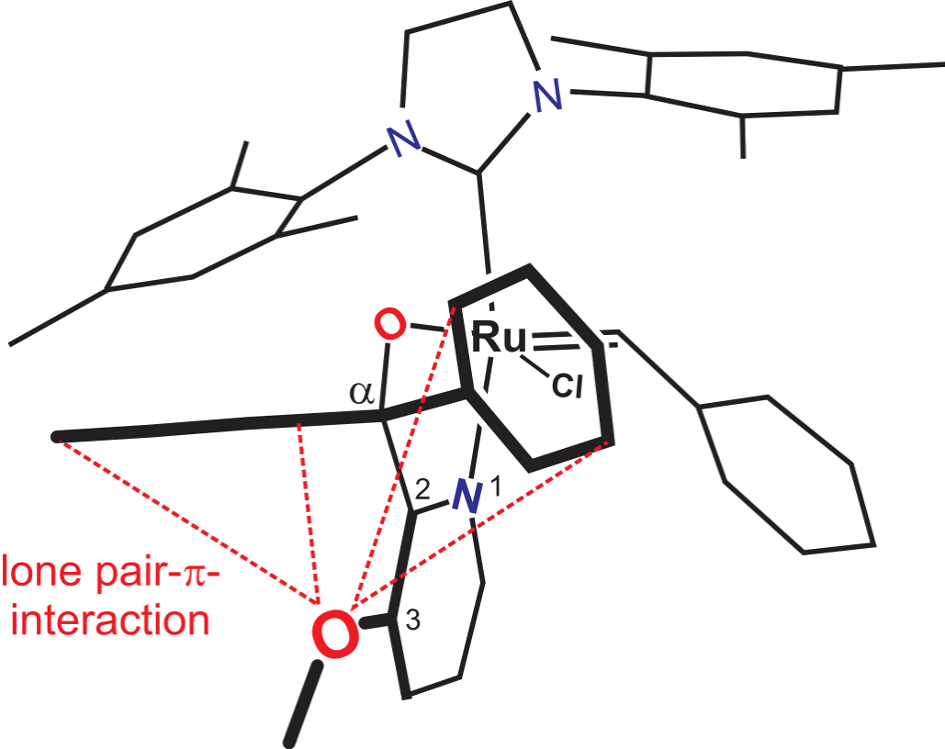
An illustration of the envisaged methoxy oxygen lone pair-aromatic π-electron interaction.

Precatalyst **6** showed the lowest activity of all precatalysts in the specified temperature ranges. It is also worthwhile to note that increasing the reaction temperature showed a significant increase in the activity of **6**. Precatalyst **9,** on the other hand, showed better performance at high temperatures (≥70 °C) compared to **6**. This may be explained by the longer Ru–N bond (2.181 Å) in the geometry-optimised structure (Figure 9) of precatalyst **9** compared to that of the Ru–N bond (2.166 Å) of **6**. A longer bond suggests a weaker Ru–N chelation thus a more active hemilabile complex. The difference in the Ru–N bond length may be attributed to the electron-withdrawing inductive effect of the OMe group making the Ru–N chelation weaker. Furthermore, a type of orbital interaction between the oxygen of the 3-OMe group and the two α-phenyl rings, i.e., an oxygen lone pair-aromatic π interaction illustrated in Figure 10, may add to the inductive effect. The longer Ru–O bond (2.031 Å), shorter C_α_–O bond (1.420 Å) and C_α_–C_2_ bond (1.541 Å) observed in precatalyst **9** when compared to the corresponding bonds in **6**, i.e., 2.028, 1.425 and 1.544 Å, respectively, supports such a premise. It may also be a plausible explanation for the envelope geometry of the five-membered ruthenacycle. In addition, the relatively low ruthenium metal positive charge on **9** would cause it to have a high initiation rate constant [19].

On the other hand, the 3-Me group in **6** will strengthen the Ru–N chelation via inductive electron-donation and steric repulsion between the methyl group and the two phenyl rings. As a result of the steric interaction **6** has a planar five-membered ruthenacycle geometry (Figure 9). In the absence of substituents on the pyridinyl moiety it is expected that the resulting precatalyst will be more active at lower temperatures. This is indeed the case when **5d** is used as catalyst.

As we have discussed earlier, the 4-Me-substituted precatalyst **7** has shown better catalytic performance in all temperatures under investigation except at 80 °C. The reason for this is that the Me group is, relatively speaking, further removed from the pyridine nitrogen so that the inductive electron-donation by the methyl group cannot significantly influence the electron density on the pyridine nitrogen. There is also no steric effect that would interfere with the Ru–N bond strength. The strengthening effect on the Ru–N chelation would, therefore, possibly be low compared to the other precatalysts.

If this is a plausible explanation for the relatively better performance of the 4-Me-substituted precatalyst **7**, one might ask what about the difference between the 3-Me-, **6**, and 5-Me-substituted, **8**, precatalysts that are at the same distance from the pyridine nitrogen? In the optimised structure of **6**, the Me group is in a crowded environment due to its proximity to the two α-phenyl groups, which upon opening the Ru–N chelation, would even become more sterically crowded. This results in a planar geometry of the five-membered ruthenacycle while **8** exhibits an envelope geometry. The Ru–N (2.179 Å) bond length in **8** is longer and the C_α_–O (1.417 Å) and C_α_–C_2_ (1.532 Å) bonds are shorter than the corresponding bonds in **6**.

In order to overcome the combined effect of the resistance that resulted from the steric crowdedness and the inductive electron-donation by the 3-Me group and open the strong Ru–N chelation, it needs relatively high energy. In **8** the methyl group is in exactly the opposite orientation to the two α-phenyl groups. Therefore, the steric crowdedness that is observed in **6** that will lead to steric resistance to open the Ru–N chelation does not exist. Thus **8** is more susceptible to hemilability than **6** and exhibits higher activity. Therefore, for 4-Me- and 5-Me-subtstituted precatalysts, only the inductive electron-donation effect of the methyl group is the reason for the increased stability. In the 3-Me- and 3-OMe-substituted precatalysts, however, the steric effect and orbital interactions work towards the stability of the precatalyst in addition to inductive effects.

### Stability of precatalysts

**Figure 11:**
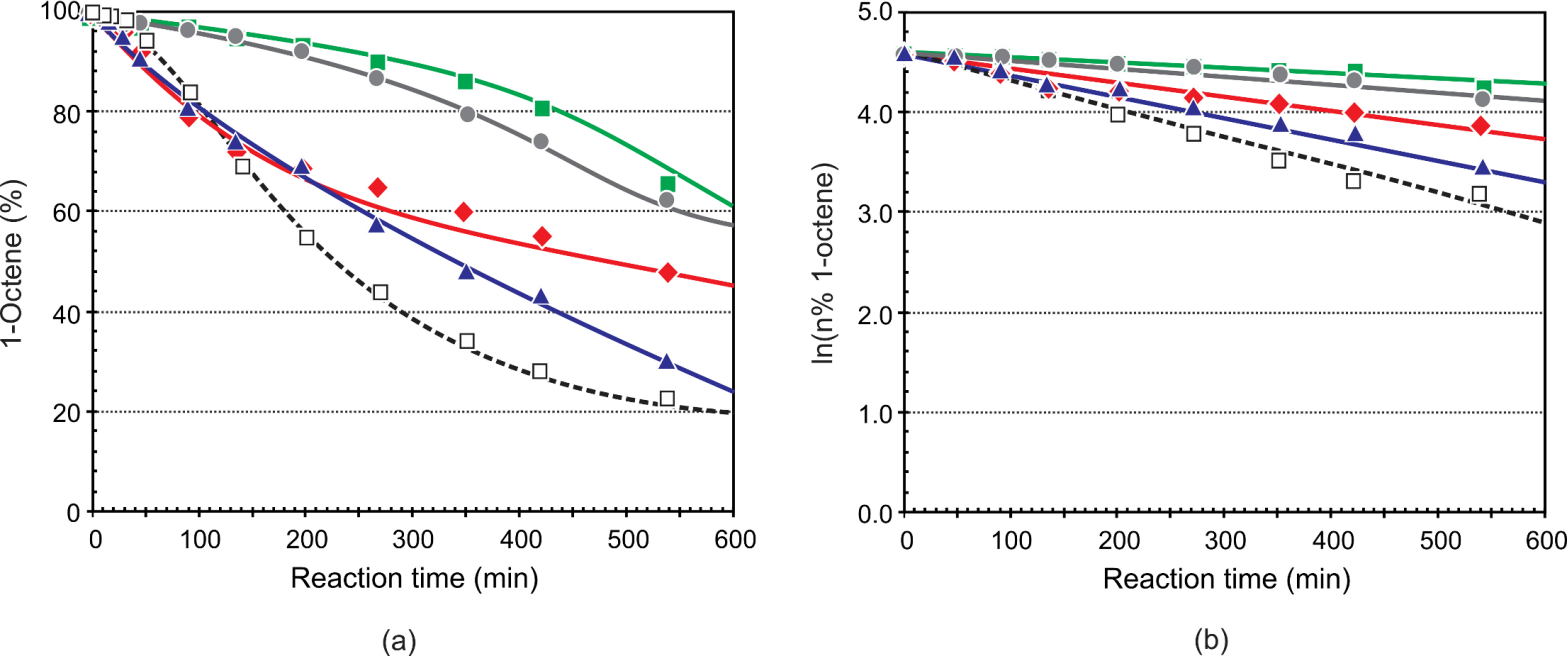
Influence of precatalysts **6–9** and **5d** on the (a) conversion of 1-octene and (b) ln([*n*%1-octene]) versus time plots (Ru/1-octene = 1:9000; 80 °C). [■ **6**, 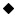 **7**, ▲ **8**, ● **9**, □ **5d**]

In previous studies [11–12,14] we investigated the stability of pyridinyl-alcoholate Grubbs-type precatalysts as seen in the improved catalytic lifetimes of these complexes. Plots of ln([starting material]) versus time, proposed by Grubbs and co-workers [20], were used as a measure of the stability of the precatalyst, i.e., a linear plot indicates a reaction with pseudo-first order rate kinetics, while a curved plot points towards catalyst decomposition. We used the conversion of 1-octene at a Ru/1-octene molar ratio of 1:9000 and a reaction temperature of 80 °C to compare the stability of **5d** with that of **5i**, **5j** and **5k** [14]. In Figure 11 the literature data (% 1-octene conversion and ln(% 1-octene)) of **5d** is compared with that of precatalysts **6** – **9** at a Ru/1-octene molar ratio of 1:9000 and a reaction temperature of 80 °C over 540 min.

The overall activity order of the catalysts follows the order **5d** > **8** > **7** > **9** > **6** up to ca. 540 min. The order **8** > **5d** 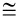 **7** > **9** > **6** is observed for both the overall metathesis and the PMPs formation. All the precatalysts exhibits first-order kinetics over the first ca 540 min when the ln(% 1-octene) plots (Figure 11b) are considered. The substituted precatalysts show better stability than **5d**, thus longer lifetimes, with **6** and **9** the slowest and **8** close to but slower than **5d**.

It is interesting to note that the stability of **5j** and **5k** correlates very well with that of **7**, while **5i** is more stable than **7** but less than **9** (comparison of current results with results in [14]). This clearly indicates that a substituent on one of the α-phenyl groups or the pyridinyl moiety has a stabilising effect on the corresponding precatalyst with a substituent on the 3-position (**6** and **9**) of the pyridinyl rendering the precatalyst the most stable. The latter two is also active at higher temperatures.

Table 6 presents the overall catalytic performance of precatalysts **5d**, and **6**–**9** at a Ru/1-octene molar ratio of 1:9000, 80 °C and 420 min. According to these results precatalyst **5d** shows the highest PMPs, TON and TOF. Although it has relatively high SMPs compared to most of the precatalysts, its overall performace prevails over the other precatalysts. The second best performance was observed for **8**, as it resulted in relatively high PMPs, TON and TOF compared to the rest of the precatalysts, although its SMPs ranks as first. The rest of the precatalysts can be ranked in a decreasing order of activity of **7** > **9** > **6**. It is clear from the data in Table 6 that the unsubstituted precatalyst **5d** is more active compared to the substituted precatalysts at 420 min. This will only be due to the substituent effect on the activity of the precatalyst.

**Table 6:** Summary of catalytic performances of different precatalyst (Ru/1-octene molar ratio 1:9000, 80 °C, 420 min).

Entry	Precat.	Conv.^a^	PMPs^a^	SMPs^a^	IPs^a^	S^b^	TON^c^	TOF^d^

1	**5d**^e^	71.2	68.0	3.0	0.3	96	6120	24.29 × 10^−2^
2	**8**	65.4	60.5	4.1	0.8	92	5445	21.61 × 10^−2^
3	**7**	44.4	43.1	1.1	0.2	97	3879	15.39 × 10^−2^
4	**9**	25.7	24.0	1.4	0.3	93	2160	8.57 × 10^−2^
5	**6**	19.4	16.4	2.3	0.7	85	1476	5.86 × 10^−2^

^a^Conversion or yield in mol %; ^b^S (selectivity) in percent toward PMPs; ^c^TON (turnover number) = [%PMPs × (Oct/Ru)]/100; ^d^TOF (turnover frequency) = TON/time in s; ^e^See reference [14].

### Effect of catalyst concentration

Earlier studies indicated that 80 °C is the optimum temperature for **5d** [10–11]. It was therefor decided to investigate the effect of the concentration of the precatalyst on the metathesis of 1-octene at 80 °C. Precatalyst **8** was chosen for this investigation at Ru/1-octene molar ratios of 1:6000, 1:9000, 1:10000 and 1:15000.

Table 7 presents the overall catalytic performance of precatalyst **8** at different Ru/1-octene molar ratios, 80 °C and 420 min. With a decrease in precatalyst concentration a direct relationship was observed with the conversion of 1-octene and PMPs, they all decreased, while the TON and TOF increased. The SMPs and IPs did not follow a specific trend while the selectivity remained the same, i.e., 92%, at all the concentrations.

**Table 7:** Summary of the catalytic performance of precatalyst **8** present in different concentrations (80 °C, 420 min).

Entry	C_8_:Ru	Conv.^a^	PMPs^a^	SMPs^a^	IPs^a^	S^b^	TON^c^	TOF^d^

1	6000	76.9	70.7	5.9	0.4	92	4240	16.83 × 10^−2^
2	9000	65.4	60.5	4.1	0.8	92	5445	21.61 × 10^−2^
3	10000	60.2	55.4	4.5	0.4	92	5539	21.98 × 10^−2^
4	15000	52.8	48.4	3.8	0.7	92	7254	28.78 × 10^−2^

^a^Conversion or yield in mol %; ^b^S (selectivity) in percent toward PMPs; ^c^TON (turnover number) = [%PMPs × (Oct/Ru)]/100; ^d^TOF (turnover frequency) = TON/time in s.

### ^1^H NMR investigation of precatalyst **7** and **5d**

Proton nuclear magnetic resonance spectrometry (^1^H NMR) is a powerful tool to study ruthenium alkylidene complexes and was used to study 1-octene metathesis in the presence of **1** and **2** [10,21–22]. The conversion of the benzylidene, [Ru]=CHPh, to the heptylidene, [Ru]=CHC_6_H_13_, and methylidene, [Ru]=CH_2_, (where [Ru] = RuL_2_Cl_2_) could be clearly distinguished using the carbene-H_α_ signals; they appeared as a singlet, triplet and singlet in the δ 18.5–20.2 ppm region, respectively. We also investigated **5a** and observed five carbene-H_α_ signals attributing three to the alkylidene species when the pyridinyl-alcoholato ligand was in the “closed” (coordinated) position; δ_C_*_H_*_Ph_ 18.05 ppm, δ_C_*_H_*_Hx_ 16.71 ppm and δC*H*H 16.08 ppm [10]. The other two was attributed to the benzylidene (δ_C_*_H_*_Ph_ 19.48 ppm) and methylidene (δ_C_*_H_*_H_ 19.76 ppm) species in the “open” (uncoordinated) position with the uncoordinated heptylidene signal not appearing probably due to the fast reaction of this species. Four signals at δ 9.48 ppm (for the coordinated ligand), 9.05 ppm, 9.22 ppm, and 9.71 ppm attributed to the H_α_ signals of the pyridine ring were also observed. The latter three signals overlapped too much to be useful.

**Figure 12:**
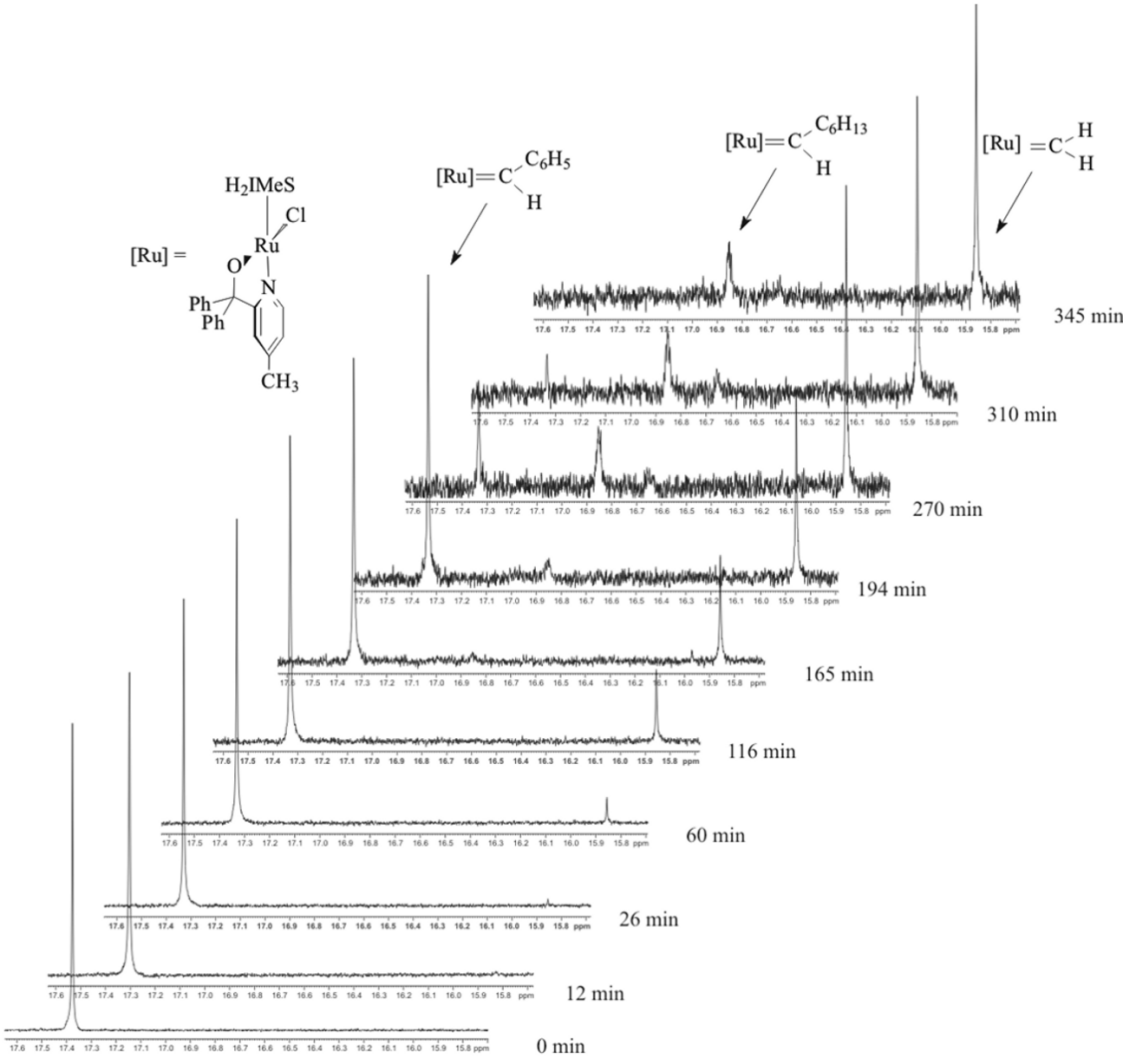
^1^H NMR spectra of the carbene-H_α_ region at different time intervals of the 1-octene/**7** reaction mixture in toluene-*d*_8_ at 90 °C.

We performed a ^1^H NMR investigation of the metathesis of 1-octene by precatalyst **7** in the temperature region 60–90 °C in order to gain some insight into the reaction mechanism. The carbene-H_α_^1^H NMR signals at 90 °C over a period of 345 min are presented in Figure 12. Three signals attributed to the benzylidene (δ 17.33 ppm, singlet), heptylidene (δ 16.85 ppm, triplet) and methylidene (δ 15.68 ppm) were observed. A small signal at δ 16.66 ppm appeared at 270 min and was not assigned (inter alia multiplicity not discernable). A different development of carbene signals over time is observed than what was reported before for **5a** [10], i.e., the methylidene signal starts to appear at 12 min while the heptylidene signal only starts to appear at 165 min. The benzylidene signal rapidly declines after 194 min and is not observed at 345 min. No clear indication of an “open” or “closed” complex was observed, so it assumed that the signals represent the “closed” species. It can be concluded that the alkylidene species of the pyridinyl-alcoholato Grubbs 2-type precatalysts are quite stable at high temperatures explaining the activity of these precatalyst at high temperatures and the slow rate of disappearance/formation of these signals confirms the longer lifetimes observed in the catalytic reactions.

**Figure 13:**
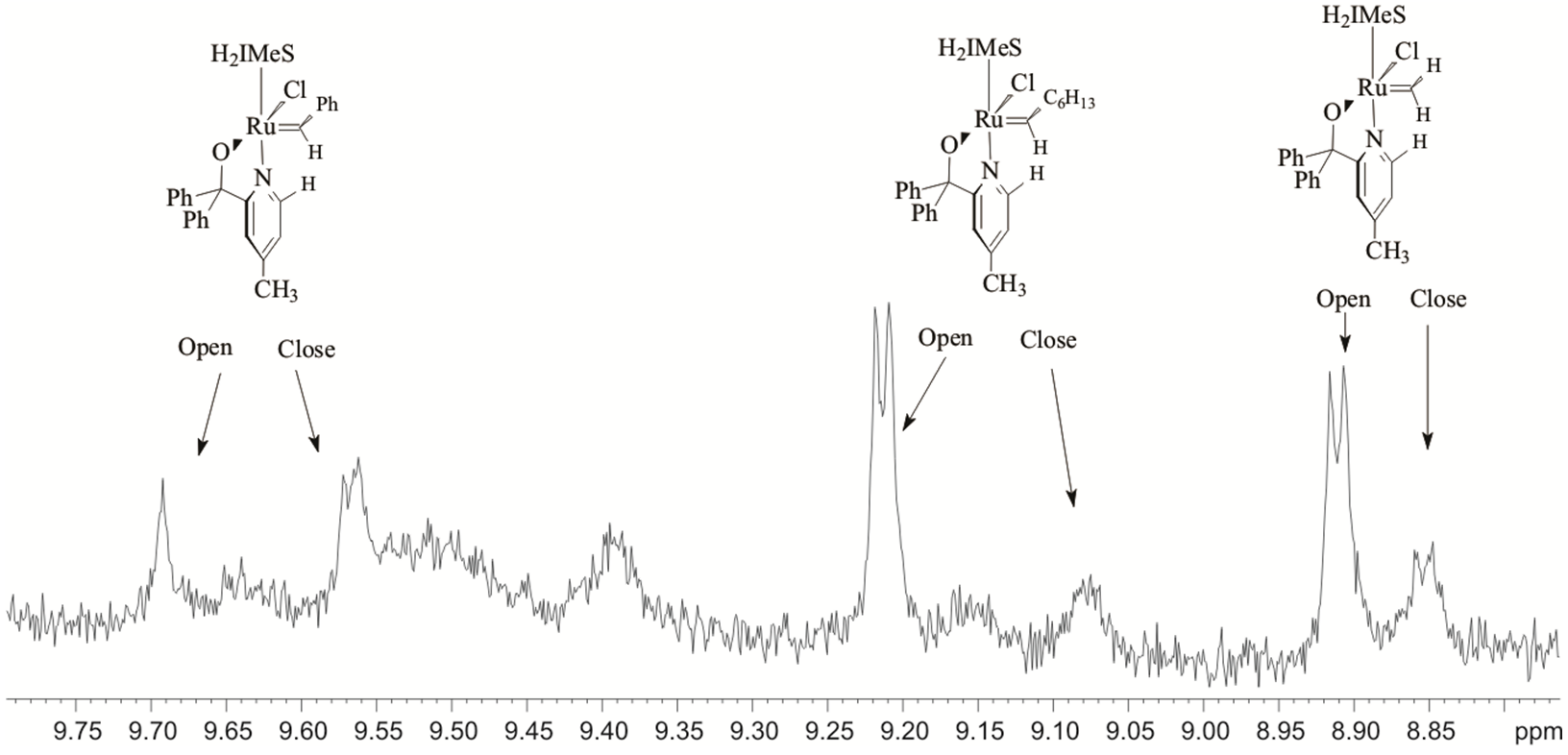
^1^H NMR spectra of the H_α_ region of the pyridine ring of the 1-octene/**7** reaction mixture in toluene-*d*_8_ at 90 °C at 345 min.

The H_α_ pyridine ring ^1^H NMR signals at 90 °C at 345 min are presented in Figure 13. Five H_α_ signals of the pyridine ring that are not observed at the beginning of the reaction were observed at δ 9.57 ppm (doublet), δ 9.22 ppm (doublet), δ 9.08 ppm (unknown multiplicity), δ 8.91 ppm (doublet) and δ 8.85 ppm (doublet). The signal at δ 9.70 ppm (singlet) was the only signal observed at the beginning of the reaction. These signals is probably due to the “open” and “closed” pyridinyl-alcoholato ligands of alkylidene species and a possible assignment is shown in Figure 13. Further research is required to gain a comprehensive understanding of the operation of the active species of the pyridinyl-alcoholato ruthenium alkylidene precatalysts.

### Effect of solvent on 1-octene metathesis using precatalyst **7**

Because toluene-*d*_8_ was used in the ^1^H NMR study it was decided to investigate if toluene as solvent has any effect on the 1-octene metathesis reaction using precatalyst **7**. Results of this investigation are presented in Table 8.

**Table 8:** Summary of the catalytic performance of precatalyst **7** present in the presence of toluene as solvent (Ru/1-octene = 1:9000, 90 °C, 420 min).

Entry	Solvent	Conv.^a^	PMPs^a^	SMPs^a^	IPs^a^	S^b^	TON^c^	TOF^d^

1	neat	81.4	72.4	8.0	1.0	89	6516	25.86 × 10^−2^
2	toluene^e^	79.8	61.8	16.9	1.2	77	5564	22.08 × 10^−2^

^a^Conversion or yield in mol %; ^b^S (selectivity) in percent toward PMPs; ^c^TON (turnover number) = [%PMPs × (Oct/Ru)]/100; ^d^TOF (turnover frequency) = TON/time in s; ^e^*V*_toluene_ = 4 mL.

An increase in SMPs formation is the only difference that was observed when toluene was used as solvent with an 8.9% increase at 420 min. This affected the other performance indicators, i.e., PMPs, S, TON and TOF; lower values than the neat reactions were obtained. The results suggest that no significant solvent effect appears to exist .However, the increase in SMPs (associated with an increase in IPs) indicates decomposition of the precatalyst to active isomerisation species, probably metal hydride species. In our NMR study no indication of the existence of metal hydride species was found.

## Conclusion

The aim of our research is to control the Ru–N bond strength of the bidentate hemilabile pyridinyl-alcoholato ligands in precatalyst **5d** in an attempt to synthesise a precatalyst with high performance for linear alkene metathesis at high temperatures. To reach this aim, we synthesised ruthenium alkylidene precatalysts by substituting one of the hydrogens of the pyridine ring of the bidentate pyridinyl-alcoholato ligand by Me and OMe groups. We synthesised the 3-, 4-, and 5-methyl and 3-methoxy-substituted **5d** precatalysts. The catalytic activity, selectivity and stability results of the Me- and OMe-substituted **5d** precatalysts, in 1-octene metathesis, showed promising results at high temperatures. The high stability, very good activity, selectivity, TON and TOF of the four precatalysts, at high temperatures, proved that the hemilability of the bidentate hemilabile pyridinyl alcoholato ligand can be influenced by monosubstitution on the pyridinyl moiety. A Ru/1-octene precatalyst concentration of 1:9000 and 80 °C were found to be the best reaction conditions for the precatalysts. Although **8** performed better than the rest of the precatalysts at 80 °C, **7** showed the best performance in the other temperatures under investigation. ^1^H NMR spectrometry was used to investigate precatalyst **7** and the active alkylidene species, i.e., benzylidene, heptylidene and methylidene, were observed. NMR evidence of the hemilabile nature of these precatalysts was found in the H_α_ region of the pyridine ring of the pyridinyl alcoholato ligand.

## Experimental

### Instruments and reagents

**^1^H NMR** (600 MHz) spectra were obtained using a Bruker Ultrashield Plus 600 Avance III spectrometer.

**GC/FID:** The progress of the metathesis reactions was followed on an Agilent 6890 gas chromatograph equipped with an Agilent 7683 auto sampler, HP-5 5% phenyl methyl siloxane capillary column and a flame ionisation detector (FID). The following general GC settings were used: Column: HP-5, 30.0 m × 320 μm × 0.25 μm, nominal; detector: FID at 250 °C; H_2_ flow rate: 40 mL/min at 20 °C; air flow rate: 450 mL/min at 20 °C; inlet temperature: 200 °C, 60.6 kPa; N_2_ carrier gas flow rate: 45 mL/min at 20 °C; injection volume: 2 µL (auto injection); syringe size 10.0 μL; split ratio: 50.4:1; split flow 94.3 mL/min; oven programming: 60 °C for 5 min; 60 to 110 °C at 25 °C/min; 110 °C hold for 10 min; 110 to 290 °C at 25 °C/min; 290 to 300 °C at 25 °C for 5 min.

**GC/MSD** analyses was performed on an Agilent 6890 gas chromatograph equipped with an Agilent 7683B autosampler, HP-5 capillary column and an Agilent 5973 mass selective detector (MSD). The oven programme was used with either a two-minute solvent delay or no solvent delay. Helium was used as carrier gas with a 1.5 mL/min flow rate at 20 °C. The following general GC settings were used: Column: HP-5, 30.0 m × 320 μm × 0.25 μm; Split ratio: 0.1:1; Split flow: 0.1 mL/min; Inlet: 250 °C, 16.6 kPa; Injection volume: 0.2 μL; Detector: 50–550 Dalton mass range; scan speed of 2.94 seconds per decade; oven programming: 60 °C (hold time 2 min); 60 to 110 °C at 25 °C/min; 110 °C (hold time 10 min); 110 to 290 °C at 25 °C/min (hold time 16 min).

**Reagents:** 2-Bromo-3-methylpyridine (95%), 2-bromo-4-methylpyridine (97%), 2-bromo-5-methylpyridine (98%), 2-bromo-3-methoxypyridine (97%), *n*-BuLi (2.5 M in hexane), benzophenone (99%), 2-*N,N*-dimethylaminoethanol (≥ 99.5%), Grubbs 2 (**2**) (97%), 1-octene (99% GC), nonane (reagent plus 99%) and toluene (99%) were purchased from Sigma-Aldrich. Toluene-*d*_8_ (99.5%) was purchased from MERCK and *tert*-butyl hydrogen peroxide (80%) from Riedel-de Haen. Diethyl ether and THF were dried over Na in the presence of benzophenone. Pentane was distilled over CaH_2_ in an inert atmosphere before using as solvent. 2-*N,N*-Dimethylaminoethanol and *n*-hexane were dried over molecular sieves (4 Å) and kept under nitrogen before use. Gas-tight Hamilton syringes were used to add reagents and dried solvents to the reactor. Acrodisc Premium 25 mm syringe filter with GxF/0.45 µm GHP membrane (PALL) was used to filter the lithium salt from the precatalyst.

### Experimental procedures

**Scheme 2:**
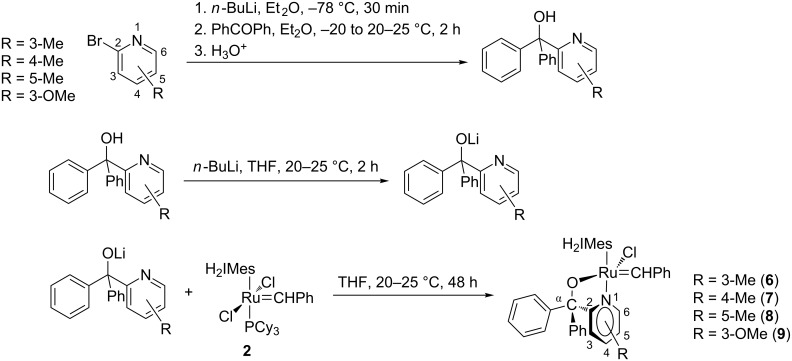
Synthesis of pyridinyl-alcohol ligands and Grubbs 2-type pyridinyl-alcoholato complexes.

**Precatalyst synthesis:** The well-established methods of Herrmann et al. [23] and Van Der Schaaf et al. [7] were used to synthesize precatalysts **6**–**9**. This is illustrated in Scheme 2.

**Benzylidene chloro(1,3-bis(2,4,6-trimethylphenyl)-2-imidazolidinylidene)[1-(3′-methyl-2′-pyridinyl)-1,1-diphenylmethanolato]ruthenium (6):** Yield 0.422 g, 89%, green powder, decomp.: 190 °C, ^1^H NMR (600 MHz, CDCl_3_) δ 16.89 (s, 1H, H of Ru=CHPh), 9.70 (t, *J* = 3.8 Hz, 1H, H-6 of C_5_H_3_N), 7.24 (m, 1H, *para* H of Ru=CHPh), 7.24 (m, 2H, *meta* H of Ru=CHPh), 7.15 (m, 2H, *ortho* H of Ph), 7.05/7.28 (m, 4H, *meta* H of mesityl), 7.00 (m, 1H, H-4 of C_5_H_3_N), 6.90 (s, 2H, *para* H of Ph), 6.97 (d, *J* = 3.7 Hz, 2H, *ortho* H of Ru=CHPh), 6.49 (s, 1H, H-5 of C_5_H_3_N), 6.75 (s, 2H, *meta* H of Ph), 4.01–3.87 (m, 4H, H of NHC), 2.56/2.18/2.11 (3 × s, 3 × 6H, H of 6-CH_3_ on mesityl), 1.18 (s, 3H, H of CH_3_ on C_5_H_3_N); MALDI–MS (*m*/*z*): [M]^+^ 807.2646 (C_47_H_48_ClN_3_ORu); IR (in cm^−1^): *v*(OH, moisture) = 3386, *v*(=C-H) = 3054, 3018, 776, *v*(CH_3_) = 2921, 2852, 1396, *v*(C=N) = 1604, *v*(C=C) = 1584–1443, *v*(C-N) = 1254, *v*(C-O) = 1157; ^13^C NMR (150 MHz) δ 290.4, 214.5, 169.9, 151.2, 148.4, 144.6, 144.4, 139.2, 137.8, 132.4, 128.8, 128.7, 126.8, 126.7, 121.3, 93.7, 51.2, 21.0, 19.1, 18.8.

**Benzylidene chloro(1,3-bis(2,4,6-trimethylphenyl)-2-imidazolidinylidene)[1-(4′-methyl-2′-pyridinyl)-1,1-diphenylmethanolato]ruthenium (7):** Yield 0.456 g, 96%, green powder, decomp.: 190 °C, ^1^H NMR (600 MHz, CDCl_3_) δ 17.09 (s, 1H, H of Ru=CHPh), 9.42 (d, *J* = 5.9 Hz, 1H, H-6 of C_5_H_3_N), 7.27 (m, 1H, H-3 of C_5_H_3_N), 7.27 (m, 2H, *para* H of Ru=CHPh), 7.24 (s, 2H, *meta* H of Ru=CHPh), 7.24/7.04 (m, 4H, *meta* H of mesityl), 7.10 (m, 4H, *ortho* H of Ph), 6.96 (t, *J* = 7.6 Hz, 2H, *para* H of Ph), 6.92 (s, 2H, *ortho* H of Ru=CHPh), 6.76 (s, 1H, H-5 of C_5_H_3_N), 6.63 (s, 4H, *meta* H of Ph), 4.04–3.90 (m, 4H, H of NHC), 2.60/2.25/2.19 (3 × s, 3 × 6H, H of 6-CH_3_ on mesityl), 1.99 (s, 3H, H of CH_3_ on C_5_H_3_N); IR (in cm^−1^): *v*(OH, moisture) = 3393, *v*(=C-H) = 3052, 3018, 755, *v*(CH_3_) = 2919, 2850, 1290, *v*(C=N) = 1611, *v*(C=C) = 1448–1443, *v*(C-N) = 1258, *v*(C-O) = 1248; ^13^C NMR (150 MHz, CDCl_3_) δ 291.7, 214.7, 170.6, 151.5, 149.8, 146.2, 143.8, 139.2, 137.8, 136.7, 129.0, 128.5, 126.8, 126.6, 122.3, 93.0, 51.3, 21.0, 19.1, 18.8.

**Benzylidene chloro(1,3-bis(2,4,6-trimethylphenyl)-2-imidazolidinylidene)-[1-(5′-methyl-2′-pyridinyl)-1,1-diphenylmethanolato]ruthenium (8):** Yield 0.304 g, 64%, dark-green crystalline powder, decomp.: 125 °C, ^1^H NMR (600 MHz, CDCl_3_) δ 17.07 (s, 1H, H of Ru=CHPh), 9.37 (s, 1H, H-6 of C_5_H_3_N), 7.26 (m, 1H, *para* H of Ru-CHPh), 7.24 (s, 4H, *meta* H of mesityl), 7.14 (m, 1H, H-3 of C_5_H_3_N), 7.10–7.02 (m, 4H, *ortho* H of Ph), 6.97 ( t, *J* = 7.5 Hz, 2H, *meta* H of Ru=CHPh), 6.92 (s, 2H, *ortho* H of Ru=CHPh), 6.92 (s, 1H, H-4 of C_5_H_3_N), 6.75 (t, *J* = 7.6 Hz, 2H, *para* H of 2Ph), 6.68/6.58 (2d, *J* = 7.3 Hz, 4H, *meta* H of 2Ph), 4.00–3.95 (m, 4H, H of NHC), 2.60/2.26/2.19 (3 × s, 3 × 6H, 6-CH_3_ of mesityl), 2.17 (s, 3H, H of CH_3_ on C_5_H_3_N); MALDI–MS (*m*/*z*): [M]^+^ 807.2660 (C_47_H_48_ClN_3_ORu); IR (in cm^−1^): *v*(OH, moisture) = 3391, *v*(=C-H) = 3055, 3020, 755, *v*(CH_3_) = 2920, 2849, 1377, *v*(C=N) = 1605, *v*(C=C) = 1481–1410, *v*(C-N) = 1261, *v*(C-O) = 1163; ^13^C NMR (150 MHz, CDCl_3_) δ 291.2, 214.5, 168.2, 151.6, 149.9, 146.3, 143.9, 139.2, 137.3, 134.9, 129.0, 128.5, 126.8, 126.5, 121.7, 92.9, 51.3, 20.9, 19.1, 18.8.

**Benzylidene chloro(1,3-bis(2,4,6-trimethylphenyl)-2-imidazolidinylidene)-[1-(3′-methoxy-2′-pyridinyl)-1,1-diphenylmethanolato]ruthenium (9):** Yield 0.465 g, 96%, green powder, decomp.: 197 °C, ^1^H NMR (600 MHz, CDCl_3_) δ 16.97 (s, 1H, H of Ru=CH), 9.38 (d, *J* = 5.5 Hz, 1H, H-6 of C_5_H_3_N), 7.30 (m, 1H, *para* H of Ru=CHPh), 7.29 (m, 2H, *meta* H of Ru=CHPh), 7.25 (m, 4H, *ortho* H of 2Ph), 7.16 (m, 1H, H-4 of C_5_H_3_N), 6.91/6.54 (s, 4H, *meta* H of mesityl), 7.04 (m, 1H, H-4 of C_5_H_3_N), 6.99 (m, 2H, *para* H of 2Ph), 6.99 (m, 1H, H-5 of C_5_H_3_N), 6.73 (m, 4H, *meta* H of 2Ph), 6.73 (m, 2H, *para* H of 2Ph), 6.43 (d, *J* = 7.7 Hz, *ortho* H of Ru=CHPh), 4.01–3.89 (m, 4H, H of NHC), 2.57/2.19/2.16 (3 × s, 3 × 6H, 6-CH_3_ of mesityl), 2.90 (s, 3H, H of OCH_3_); MALDI–MS (*m*/*z*): [M]^+^ 823.2417 (C_47_H_48_ClN_3_O_2_Ru); IR (in cm^−1^): *v*(OH, moisture) = 3388 *v*(=C-H) = 3054, 3015, 755, *v*(CH_3_) = 2920, 2848, 1316, *v*(C=N) = 1601, *v*(C=C) = 1586–1457, *v*(C-N) = 1258, *v*(C-O) = 1230; ^13^C NMR (150 MHz, CDCl_3_) δ 290.5, 214.5, 162.9, 151.5, 151.4, 149.1, 142.3, 139.2, 137.3, 137.2, 129.0, 128.6, 126.8, 122.1, 93.6, 55.4, 51.3, 20.9, 19.1, 18.8.

**Metathesis reactions:** The metathesis reactions were carried out in 5 mL small-scale glass reactors. The reactor containing a small magnetic bar was flushed with nitrogen and an appropriate amount of precatalyst added by weighing. Once again, the contents of the reactor were carefully flushed with nitrogen and the reactor was sealed. The sealed reactor was placed in an aluminium block on a magnetic stirrer. The temperature was set to the desired temperature and allowed to stabilise prior to the reactor being placed in the block. The temperature was regulated throughout the reaction using a temperature controller fitted with a thermocouple. After one minute of heating nonane (0.25 mL) was added via gastight syringe (1 mL) as an internal standard, followed by the addition of 1-octene (5 mL) via gastight syringe (5 mL). Samples (0.1 mL) were withdrawn at time intervals for ca*.* 520 min with a gastight syringe (1 mL), transferred to a GC vial (1 mL), quenched with toluene (0.3 mL) and *tert*-butyl hydrogen peroxide (2 drops), and then injected into a GC/FID by auto sampler. The metathesis reaction was terminated after 1440 minutes and analysed by GC/FID. Some samples were also analysed by GC/MSD. Each experiment was repeated at least three times.

**^1^H NMR investigation of metathesis reaction:** An NMR tube was placed in a Schlenk tube, evacuated with a vacuum pump and then flushed with a stream of argon. The same procedure was repeated and then 12 mg (0.015 mmol) of precatalyst **7** was added to the NMR tube. Once again, the contents of the NMR tube were flushed with argon and toluene-*d*_8_ (0.65 mL) added. The catalyst was dissolved by shaking the contents and 1-octene (0.1 mL, 0.64 mmol) added immediately before putting the tube in the spectrometer for temperature ranges 30–50 °C. ^1^H NMR spectra were recorded at 5–6 minute intervals for 5–8.5 h. For temperature ranges 60–90 °C, ^1^H NMR of the precatalyst was done alone before adding the 1-octene. The precatalyst (11.5 mg, 0.014 mmol) and anthracene (5.2 mg, 0.03 mmol) were mixed in the metathesis reaction where anthracene was used as an internal standard.

### Computational details

**Geometry optimisation:** Geometry optimisation of the precatalysts was done using the DFT module DMol^3^ of Materials Studio 6.1. The generalized gradient approximation (GGA) with a double numerical basis set and a p-function (DNP) was used. The exchange correlation functional PW91 was investigated. All electrons were treated explicitly and the net charge of all the structures was set to zero. Energies were calculated with frequencies using coarse-grained parallelisation in order to avoid optimised structures with negative frequencies.

**Atomic charge calculation:** Total electron density was calculated with fine grid resolution, 0.15 Å grid interval and 3.0 Å border. Mulliken atomic charges of Ru were calculated from population analysis and total electron density.

**Ru-atom bond length measurement:** All bond lengths were measured from the optimised complexes with α,α-diphenyl-(monosubstituted-pyridin-2-yl)alcoholato ligands.

**Hardware:** 1. Personal computer (HP); (Windows 7 Enterprise © 2009 Microsoft Service Pack 1, Intel® Core™ i5-2450M CPU @ 2.50 GHz, 2.50 GHz; 64-bit operating system).

2. HPC: 336 CPU Cluster with 1 × Master Node: (HP BL460C G6 - 2 Quad Core 2.93 GHz, 16 GB RAM, 2 146 GB HDD), 40 × Compute Nodes: (HP BL460C G6 - 2 Quad Core 2.93 GHz, 16 GB RAM, 2 146 GB HDD, ProLiant BL2 x 220c G5, HP BL460C G1), 1 × 3 TB HP EVA 4400 SAN and 1x HP BL460C G6 Storage Server, Operating system on compute nodes: Scientific Linux SL release 5.3, Cluster operating system: Rocks 5.2 - Scientific Linux SL release 5.3.
